# Discharge instructions for caregivers in the context of pediatric emergency care: a narrative synthesis protocol

**DOI:** 10.1186/2046-4053-3-26

**Published:** 2014-03-14

**Authors:** Janet A Curran, Andrea Murphy, Mandi Newton, Roger Zemek, Lisa Hartling, Amy Plint, Jill Chorney, Shannon MacPhee, Samuel G Campbell, Mona Jabbour, Darlene Boliver, David Petrie, Randy Colwell, Kate MacWilliams, Alicia Nolan

**Affiliations:** 1 IWK Health Center, Dalhousie University, 5869 University Avenue, PO Box 15000, Halifax, NS B3H 4R2, Canada; 2 College of Pharmacy, Dalhousie University, 5869 University Avenue, PO Box 15000, Halifax, NS B3H 4R2, Canada; 3 University of Alberta, 116 St. and 85 Ave, Edmonton, AB T6G 2R3, Canada; 4 Children’s Hospital of Eastern Ontario, 401 Smyth Rd, Ottawa, ON K1H 8L1, Canada; 5 Alberta Research Center for Health Evidence (ARCH), University of Alta, 116 St. and 85 Ave, Edmonton, AB T6G 2R3, Canada; 6 IWK Health Center, 5850/5980 University Avenue, PO Box 9700, Halifax, NS B3K 6R8, Canada; 7 Halifax Infirmary, 1796 Summer St, Halifax, NS B3H 3A7, Canada; 8 Halifax Infirmary (CDHA), 1796 Summer Street, Halifax, NS B3H 3A7, Canada; 9 South Shore Regional Hospital, 90 Glen Allan Drive, Bridgewater, NS B4V 3S6, Canada; 10 School of Nursing, Dalhousie University, 5869 University Avenue, PO Box 15000, Halifax, NS B3H 4R2, Canada

**Keywords:** Narrative synthesis, Discharge instruction, Patient education, Emergency medicine, Pediatrics

## Abstract

**Background:**

The period following discharge from a pediatric emergency department (ED) can be a time of significant vulnerability for caregivers who provide ongoing care to their child when they return home. Discharge communication practice varies widely at the individual practitioner and departmental level. At present, there are no nationally accepted guidelines for discharge communication for children and/or their caregivers in the ED.

The primary objective of this knowledge synthesis is to understand how and why discharge instructions work and under what conditions. We will also examine the contextual factors and barriers and facilitators associated with discharge communication across varied ED settings.

**Methods/Design:**

Using an integrated narrative approach, we will synthesize different types of evidence and explore relationships within and between included studies to develop a theory-based and knowledge user-informed discharge communication practice guideline. We will follow key principles for knowledge synthesis including: (1) involvement of a multidisciplinary team (for example, information specialists, statisticians, and content experts); (2) developing focused and answerable questions in collaboration with the knowledge users; (3) using a systematic method including specific tools and techniques appropriate for answering questions concerned with effectiveness and the implementation of interventions; and, (4) involving knowledge users throughout the process in an integrated knowledge translation approach.

**Discussion:**

This collaborative and narrative approach will be a determining factor in increasing the reliability, validity and relevance of the study findings for healthcare practice and policy decision-makers.

**Trial registration:**

PROSPERO registration number: CRD42014007106

## Background

In 2006, children under 17 years of age accounted for one in four visits to emergency departments (EDs) in Ontario, Canada [1]. Of the one million visits made in a year by 685,000 children, one in three visited more than once and one in fifteen returned to the ED within 72 hours of the index visit [1,2]. Following a visit to the ED, the majority of children (87%) are discharged to their home residence. Ideally, caregivers should leave the ED with the necessary knowledge and skills to effectively manage their child’s care at home. Optimal delivery of discharge instruction to caregivers who present to the ED with their children is not well understood and there has been a recent call to action for improved development of quality and safety indicators in the ED [3,4]. Standardized instructions given to parents upon ED discharge can improve knowledge and satisfaction with care [5]. The quality of interpersonal interaction with parents about the activities associated with their ED visit has also been shown to be important for overall satisfaction [6]. Communication between the ED providers and parents should effectively complete three tasks during the discharge process: communicate important information, verify comprehension, and tailor the discharge instruction to address areas of misunderstanding [7]. However evidence suggests this is not always the caregivers’ experience.

Recent studies demonstrate that discharge instruction practices of EDs and individual practitioners vary widely [8,9] and a number of patient, provider and environmental factors influence effectiveness of the strategies [7]. Emergency care often depends on the coordination and interaction between a number of professionals inside and outside of the ED and generally takes place in a compressed timeframe. The chaotic nature of the ED setting is also characterized by frequent workflow interruptions and the time intended for discharge communication can sometimes be borrowed or compressed to address more urgent activities [10,11]. One observational study of ED visits demonstrated that on average, the discharge process lasted for 76 seconds [12]. Comprehension of discharge communication is an important factor in preventing unnecessary return visits and adherence to discharge instructions. However, patients’ understanding of discharge information is rarely assessed [12]. Further, the majority of children seen for emergency care are treated in community EDs and cared for by physicians without specialty training in pediatric emergency medicine, therefore lending more opportunity for variability in practice.

Research has also shown that following discharge, many people are unable to list their diagnosis, recount the name and purpose of medications they received, outline the recommended post ED care or know when they are required to seek further medical advice or attention [13-15]. Further, it has been found that patients who receive discharge instructions are often unable to read or comprehend the information [16]. Poor quality discharge communication in the ED can impact important variables in healthcare utilization, such as unscheduled return visits to the ED [17,18]. Younger children and patients who present during the busiest hours are more likely to return to the ED after the index visit [2]. The lack of standards and considerable variation in practice regarding discharge instruction in EDs poses a quality and safety risk for children and parents/caregivers.

To date the literature regarding discharge instructions for caregivers of children in the ED has not been synthesized. A previous review focused on discharge instruction specific to emergency practice settings has primarily targeted adults [19]. Contributions to this review regarding discharge communication for children and caregivers are limited due to poor methodological quality of included studies and the lack of reporting of relevant outcomes for a pediatric population. The review authors conclude that the relationship between discharge communication and health outcomes needs to be characterized in future reviews. This continues to be an important gap in the discharge communication literature. We will conduct a narrative synthesis to better understand how and why discharge instructions work in the context of pediatric emergency care. This review will address the following objectives: 1) characterize studies which evaluate discharge instructions for caregivers of children in EDs; 2) collect and examine documents which outline recommendations for developing and implementing discharge instructions for caregivers in an ED setting; 3) collect and examine policies for developing and implementing discharge instructions used in Canadian EDs in order to determine the extent to which they meet criteria that are associated with positive outcomes; 4) produce recommendations for the development and implementation of discharge instructions in Canadian EDs.

## Methods/Design

Understanding how and why discharge instructions work under different conditions will require exploring a range of qualitative and quantitative research with consideration also given to practice and policy documents. A narrative synthesis process is useful for combining different types of evidence and examining relationships within and between studies and reports [20]. This approach exposes the context and characteristics of each study and the similarities and differences are then compared across studies [21].

**Figure 1:**
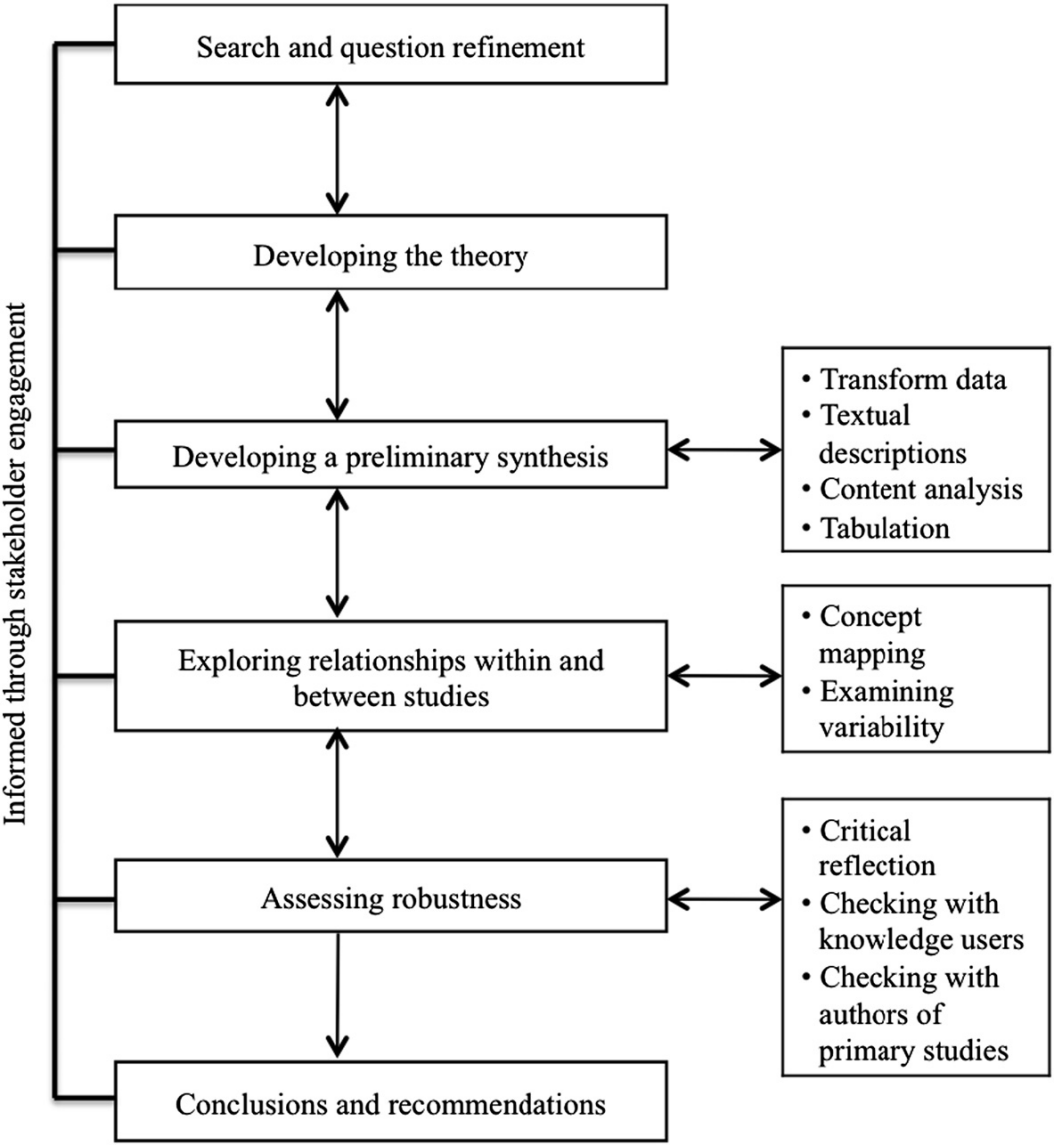
**Integrative narrative synthesis process.** Adapted from Popay J, Roberts H, Sowden A, Petticrew M, Arai L, Rodgers M, Britten N, Roen K, Duffy S: *Guidance on the Conduct of Narrative Synthesis in Systematic Reviews. A Product from the ESRC Methods Programme 2006*.

Our integrated narrative synthesis approach will be guided by methods outlined by Popay *et al*., 2006, [22] including: 1) question refinement and searching the evidence; 2) developing a theory; 3) preliminary analysis; 4) exploration of relationships; 5) assessment of robustness and; 6) conclusions and recommendations. We will follow key principles for knowledge synthesis: (1) use of a multidisciplinary approach involving information specialists, statisticians, and content experts; (2) development of focused and answerable questions at the outset in collaboration with the end users; (3) use of a systematic method for narrative synthesis including specific tools and techniques appropriate for answering questions concerned with effectiveness and the implementation of interventions; and (4) involvement of knowledge users throughout the process in an integrated knowledge translation approach in order to ensure that the questions and results will be relevant to their needs [22,23]. While our methodological description would suggest a linear process, the different stages in the synthesis will occur iteratively (Figure 1).

### Question refinement and searching for evidence

Refining the research questions: we will begin by conducting a scoping search of potentially relevant literature to determine the nature and distribution of relevant studies and other sources (for example, policies) on this topic. We will map the available literature to describe the types of interventions evaluated, the different types of implementation strategies and study designs used, and the volume of potentially relevant literature. Following the mapping exercise, our team of researchers and knowledge users will participate in a face-to-face meeting to discuss the results and refine the research questions as required.

Searching for evidence: we will work with an information specialist to conduct a comprehensive search of the published and grey literature to identify all relevant studies. Our search strategy will include a combination of formal protocol-driven strategies in health related databases (MEDLINE®, CINAHL, and EMBASE) and more informal approaches such as snowball methods and reference tracking. We will also hand-search the last five years of major emergency journals (*Annals of Emergency Medicine*, *Academic Emergency Medicine*, *American Journal of Emergency Medicine*, *Journal of Emergency Nursing*, *Pediatric Emergency Care*). The search will be peer-reviewed by a second information specialist as recommended by the recent PRESS initiative [24].

We will identify policies and recommendations that guide the development and implementation of discharge instructions for pediatric emergency care. We anticipate that these policies and recommendations will be found primarily in the grey literature (for example, institutional documents and professional organization recommendations), therefore our search will be purposive and iterative. Knowledge users will identify organizations and centers in North America and other geographic regions that would have similar healthcare practices to Canada (for example, Australia, New Zealand, Europe, the United Kingdom, and so on). We will search the websites of these organizations and centers, as well as identify individuals within the organizations or centers that may provide relevant information. We will write to the corresponding authors of included studies to identify relevant policies and best practice recommendations. We will also contact the ED Directors from the 15 pediatric emergency departments across Canada to solicit policies and/or guidelines regarding discharge communication for parents or children in the ED. Our intent is to identify policies/recommendations that will be relevant to the Canadian context.

Article screening and selection*:* we will follow recommended approaches to study selection, [23] that is, having two reviewers independently apply pre-defined inclusion criteria to all identified literature. The study selection will involve two stages. First, we will review the titles and abstracts of all citations. Second, we will retrieve the full text of all potentially relevant studies. Disagreement between the two reviewers will be resolved by a third reviewer.

Inclusion/Exclusion criteria*:* eligible studies or reports will include children less than 19 years of age or their parents, who present to the ED with any emergent or urgent clinical complaint. Examination of interventions and/or processes related to discharge instruction in an ED must be stated as the primary objective of the study. Eligible studies must report primary outcome data for children or caregivers of children separately from any adult data presented in the study. Studies focused only on an adult population or non-ED practice settings will be excluded. The criteria will be reviewed by all members of the research team and the knowledge users at the outset of the project after the research questions are finalized. Further, the criteria and forms will be pilot tested by two reviewers on a sample of five studies or reports, and revisions made accordingly with input from other co-investigators as needed.

Quality appraisal of relevant studies*:* assessing the quality of qualitative, quantitative and mixed methods studies is challenging because they represent distinct traditions with epistemological differences. Our approach to quality assessment will follow the ‘fitness of purpose’ strategy for assessing research quality for evidence based policy and practice [25]. All studies and policies will be assessed by two independent reviewers against two criteria: relevance (appropriate content for the review questions) and quality. Standardized quality appraisal tools (for example, Critical Appraisal Skills Programme) will be used depending on the research design. Quality appraisal data will be summarized in tables and where possible, we will assess for publication bias using graphical (that is, funnel plot) and statistical methods (rank correlation test, weighted regression, trim and fill method).

Data abstraction and coding*:* data will be extracted from all included studies by two independent reviewers and managed using NVivo 10 (QRS International) A comprehensive data extraction form will be developed based on the refined research questions and will be tested on a set of qualitative and quantitative reports before full application. Data extraction elements will likely include the detailed description of the intervention components, underlying theory or assumptions about causal mechanisms supporting the different intervention components, characteristics of the participants, the context in which it was introduced, guideline recommendations with corresponding levels of evidence, outcomes and reported factors and/or processes identified as impacting on implementation.

### Developing the theory

This next step in the narrative process is critical for beginning to understand what interventions included in the synthesis work, for whom and under which circumstances. Theory presents a systematic way of understanding events, behaviors or situations [26]. Revealing the theoretical underpinnings of the assumptions made about the mechanisms of actions and causal pathways is critical [27]. We will use a taxonomy of behavior change techniques developed by Abraham and Michie (2008) [28] as a guiding framework for linking the behavior change techniques described in the interventions with the relevant theory. Our goal is to begin to identify a range of theories and explanations for how discharge instruction interventions are expected to work under different conditions. We will work with the data extraction tables to correlate the underlying theory or assumptions reported by included study authors with the behavior change techniques described by Abraham and Michie [28].

### Developing a preliminary synthesis

We will develop a preliminary synthesis using a number of strategies. First, textual summaries will be developed for each of the individual quantitative and qualitative sources. We will use a systematic method to produce the narrative summaries; including reporting the same information for all studies if possible and in the same order [21]. As a minimum, summaries will include details about underlying theory, description of intervention, implementation strategy, context and setting, and outcomes. These structured summaries will elaborate and put into context the extracted data and assist with arranging reports and studies into more homogenous groups [20].

Second, data will be transformed to construct a common rubric across quantitative studies. For studies examining the effectiveness of discharge instructions, we will summarize data using either odds ratios for dichotomous outcomes or mean differences for continuous outcomes. We will quantify statistical heterogeneity using the I^2^ statistic [29] and explore sources of heterogeneity where relevant. We will assess for publication bias (where possible) using graphical (that is funnel plot) and statistical methods (rank correlation test, weighted regression, trim and fill method) [30-32]. Results will be presented using forest plots.

Third, tables will be created to provide details of study design, target population, intervention, implementation strategy, results of quality assessment, and outcome measures (including direction of effect) to allow for visual comparisons across studies. Studies will be grouped according to type of intervention, factors impacting implementation (barriers and facilitators), outcome (process or patient) and target population (caregivers only, caregivers and child).

Finally, content analysis will be carried out on data extracted from all included studies. Content analysis is a systematic technique for categorizing textual data into themes [33]. A directed approach [34] to content analysis will be used to classify data according to: 1) theories identified in the theory development phase; 2) types of interventions used in the included studies; 3) types of implementation strategies used in the included studies; 4) types of outcome measures used. A thematic analysis will then be used to identify prominent themes and subthemes [35]. This strategy of deductive and inductive coding will identify important concepts and patterns in the data and will facilitate comparison of best practice strategies with current policies and recommendations. This technique will assist with revealing trends and gaps in the evidence by exposing the dominant categories across existing policies.

### Exploring relationships within and between studies

Examining variability in outcomes, underlying theory, study design, interventions and implementation strategies and study population will draw attention to the characteristics of the different studies and reports and assist with understanding the influence of different variables [22] (for example, rural/urban settings, targeted discharge diagnosis, discipline of participants). We will develop descriptive summaries of each variable. This analytic step will allow us to uncover patterns and relationships between the study results and the key aspects of the study population, intervention and context. Characterizing the data in this way will create the opportunity to discuss study strengths and weaknesses in detail. We will use a lines-of-argument technique [36] to discuss the influence of each of the individual variables on the total body of knowledge.

We expect to find a high degree of heterogeneity in included studies; therefore we will use a concept mapping exercise to further explore relationships in the data [37]. A concept map is a knowledge representation strategy in which major concepts (represented by a word or phrase) are linked in a hierarchical structure through words or symbols. In a narrative synthesis, a concept map can provide a visual representation of the state of knowledge about a topic while providing direction for future research [38]. We will develop a preliminary concept map focused on describing the characteristics of an optimal discharge instruction for caregivers. Concepts identified in the preliminary synthesis will be organized in a hierarchical structure with the most general concepts situated at the top and the least general concepts at the bottom. Next connecting lines will be drawn to indicate a relationship between two concepts and words or phrases will be inserted on the connecting lines to illustrate how the concepts are connected. The concept map will be revised and concepts re-positioned to lend clarity to the structure based on feedback from the research team.

### Assessing robustness

We will use three techniques recommended by Popay *et al*. [22] to assess the strength of the evidence produced for drawing conclusions about best practice recommendations.

(i) Critical reflection: we will develop a process log to prospectively document team discussions at all teleconference and face-to-face meetings outlining details about input from the different stakeholders groups and underlying rationale for decisions and actions taken at each stage of the synthesis process.

(ii) Checking with the knowledge users: knowledge users will be involved in each step of the review. We will seek consensus from our knowledge user group to shape the research question, interpret the findings and develop practice and policy recommendations.

(iii) Checking with authors of primary studies: if feasible (dependent on the number of included studies) we will share the summary of key findings with authors of included studies. We will offer authors a three week timeframe to comment on the summary of findings. This will be solicited prior to the final teleconference meeting between researchers and knowledge users.

## Discussion

The quality of discharge communication can have an important impact on the healthcare system, patient outcomes and parental satisfaction. This study aims to address a critical gap in the pediatric emergency care discharge communication literature. To our knowledge, this body of literature has never been synthesized and there is an identified need to characterize the relationship between discharge communication and patient and health outcomes. This narrative review aims to further our understanding of how and why discharge communication strategies work across a range of conditions through an exploration of both quantitative and qualitative literature and an examination of existing policies. This approach we will lead to development of a framework that integrates evidence and theory. Through these linkages, we hope to map a range of theories which might be useful for explaining how discharge communication strategies work and under what conditions.

The results and recommendations will be relevant to healthcare practitioners, health administrators and planners, and ultimately to patients and their caregivers. From the inception of this review, we have used a range of knowledge users, including urban and rural practitioners, health administrators and parents to inform our work through engagement at each stage. This approach allows all stakeholders involved to develop a shared perspective on discharge communication. We believe this collaborative approach will be a determining factor in increasing the reliability, validity and relevance of study findings for healthcare policy decision-making. Ultimately, our aim is to develop recommendations that will assist decision-makers in development of best practice policies for discharge communication in pediatric emergency care settings.

A possible limitation of the proposed review relates to variation in the reporting of complex discharge communication interventions as well as the range of context and settings in which caregiver discharge communication occurs. Inclusion of different types of evidence and our strategies for assessing robustness should help to address this limitation. This review is the first step toward building a robust body of evidence to influence healthcare decision-making concerning discharge communication in a pediatric emergency care context. Recommendations arising from this review will inform future design and evaluation of discharge communication interventions for caregivers.

## Abbreviations

ED: emergency department.

## Competing interest

The authors declare they have no competing interests.

## Authors’ contributions

JAC conceived of the study, developed the study protocol and developed the first draft of this manuscript. AM participated in developing the study protocol and contributed to writing the first draft of this manuscript. MN participated in developing the study protocol and editing the first draft of this manuscript. LH participated in developing the study protocol. RZ participated in developing the study protocol and editing the first draft of this manuscript, SM contributed to editing the study protocol and this manuscript. JC contributed to developing the study protocol. SC contributed to developing the study protocol. DP contributed to developing the study protocol. AP contributed to developing the study protocol and editing the first draft of the manuscript. MJ contributed to developing the study protocol. RC contributed to developing the study protocol. DB contributed to developing the study protocol. KM contributed to developing the first draft of this manuscript. AN contributed to editing the first draft of this manuscript. All authors read and approved the final manuscript.
